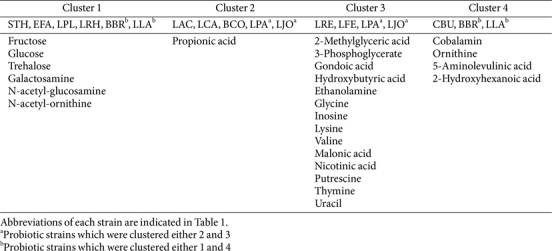# Folate Production by *Streptococcus thermophilus* IDCC 2201 and Its Impact on Human Gut Microbiota

**DOI:** 10.4014/jmb.2502.02045

**Published:** 2025-05-28

**Authors:** Eoun Ho Nam, Minjee Lee, Donggyu Kim, Young Hoon Jung, Jungwoo Yang, Minhye Shin

**Affiliations:** 1 Department of Microbiology, College of Medicine, Inha University, Incheon 22212, Republic of Korea; 2 Program in Biomedical Science and Engineering, Inha University, Incheon 22212, Republic of Korea; 3 Ildong Bioscience, Pyeongtaek-si 17957, Republic of Korea; 4 Food and Bio-Industry Research Institute, School of Food Science & Biotechnology, College of Agriculture and Life Sciences, Kyungpook National University, Daegu 41566, Republic of Korea; 5 Department of Microbiology, College of Medicine, Dongguk University, Gyeongju 38066, Republic of Korea

**Keywords:** Probiotics, *Streptococcus thermophilus*, B vitamins, folate, gut microbial community

## Abstract

Probiotics have been extensively investigated as potential food supplements for human health benefits. Metabolites derived from probiotics are the primary factors that characterize each strain’s functionality and play a crucial role in shaping their effects on the human host. In this study, we characterized the secreted metabolite profiles of sixteen commercial probiotic strains and identified *Streptococcus thermophilus* IDCC 2201 as a major folate producer. To investigate its effects on gut microbiota, *S. thermophilus* was co-cultured with individual species comprising the human gut microbial community. Specific bacteria, such as *Bacteroides thetaiotaomicron*, *Veilonella parvula*, and *Ruminococcus faecis*, grew dependently on both folate and *S. thermophilus*. These bacteria exhibited greater growth in the presence of folate than in its absence, with 2.8-, 3.6-, and 3.9-fold increases, respectively. Additionally, they showed relatively higher growth when co-cultured with *S. thermophilus* compared to other bacterial species, with 1.2-, 1.3-, and 1.9-fold increases, respectively. Our results indicate that the interaction between probiotics and the human gut microbiota can influence changes in ecological balance through nutrient cross-feeding, and understanding this interaction can be applied to precision probiotic therapies.

## Introduction

Probiotic interventions have been applied as potential therapeutics for human health, including gut dysbiosis, inflammation, and mental disorders [1-3]. Several probiotic mechanisms have been established for their health benefits: immune modulation, competition with pathogens, stimulation of gut epithelial proliferation, and production of bioactive compounds [4-7]. However, despite their effects leading to massive popularity among the public, the evidence of efficacy remains largely heterogeneous and strain-dependent. To overcome these challenges, studies on probiotics have shifted toward precision probiotics by addressing strain-dependent metabolic phenotypes and their associations with microbial communities as well as the host [8, 9].

Probiotics produce a variety of beneficial metabolites, including short-chain fatty acids (SCFAs) and vitamins, that are closely related to human health. SCFAs are volatile saturated fatty acids with fewer than six carbon atoms, produced by intestinal fermentation of indigestible carbohydrates [10]. The most common SCFAs are acetic, propionic, and butyric acids, which are utilized as energy sources in peripheral tissues and the colonic epithelium. In addition to their role in energy metabolism in the colon, SCFAs improve mucosal barrier function by promoting intestinal mucus production and colon cell proliferation [11, 12]. More recently, SCFAs have been reported to be associated with the immune system and the modulation of the gut-brain axis, affecting human health and diseases, including inflammatory bowel disease, irritable bowel syndrome, type 2 diabetes, and colon cancer [13]. Vitamins are essential micronutrients that act as coenzymes or cofactors in various cellular metabolic processes. Among the several classes of vitamins, water-soluble B vitamins play crucial roles in energy metabolism, the biosynthesis of nucleic acids and amino acids, and the maintenance of the immune system [14]. Despite the importance of these nutrients, humans lack essential genes for vitamin biosynthesis; thus, their acquisition depends entirely on dietary intake or supply from commensal gut bacteria [15].

It is well known that SCFAs and vitamins provided by gut microbiota, often by probiotics, are absorbed by the host and exert beneficial effects on health [16]. However, given that the human intestine harbors numerous bacterial species that inhabit distinct ecological niches, the effects of nutrient biosynthesis and their cross-feeding on gut microbial interactions are still not well understood. Nutrient cross-feeding is a type of interaction that involves the exchange of nutrients among different microbial species within a community [17]. This mechanism significantly affects the host by shaping community composition and the integrated metabolome of the community [17]. Among the various nutrients suitable for cross-feeding, vitamins are exceptionally well-suited for microbial interactions in the distal gut. For example, cobalamin (vitamin B_12_) mediates the growth and metabolism of different species, including *Bacteroides thetaiotaomicron*, which is a B_12_ auxotroph [18, 19]. In the termite gut, *Treponema primitia* requires folate (vitamin B9) for growth and can obtain it from other gut microbes such as *Lactococcus lactis* and *Serratia grimsesii* [20]. These cross-feeding interactions contribute to the integral function of a healthy microbiota or to disease, depending on the context. However, studies on cross-feeding have achieved limited understanding on metabolic functions due to its complexity.

In this study, we characterized metabolic signatures of 16 probiotic strains, including those from *Bacillus*, *Lactobacillus*, *Lactococcus*, *Bifidobacterium*, and *Streptococcus* genera, focusing on SCFAs and B vitamins production. Among them, *S. thermophilus* IDCC 2201 was selected as a major folate prototroph, and its effects on the changes of community distribution in the absence of folate were investigated using a pairwise co-culture experiment. This work identified metabolic features of widely used probiotics and elucidated their effects on gut microbiota with respect to folate cross-feeding, which can be applied to precision probiotic therapies.

## Materials and Methods

### Bacterial Strains and Culture Conditions

The bacterial strains used in this study are listed in Table 1. Most probiotic species were obtained from Ildong Bioscience and grown in modified Gut Microbiota Medium (GMM), which is commonly used for cultivation of a variety of gut bacterial species, at 37°C under anaerobic conditions for 24 h in a static incubator. For a synthetic human gut bacterial community, 17 species of the most common bacteria present in the human gut were obtained from the Biological Resource Center (Korean Collection for Type Cultures KCTC, Republic of Korea; Table 1) and maintained in strain-specific media under anaerobic conditions. Most strains were grown in Tryptic soy broth hemin menadione supplemented with 0.05% (w/v) L-cysteine and 5% (v/v) sheep blood (MBcell, Republic of Korea), except for *Bifidobacterium longum* *subsp.infantis*, *Lacticaseibacillus rhamnosus*, *Lacticaseibacillus casei*, *S. thermophilus*, *Bacteroides ovatus*, *Parabacteroides distasonis*, and *Akkermansia muciniphila*. *B. infantis* was grown in BL broth (BD Difco, USA), while *L. rhamnosus*, *L. casei*, and *S. thermophilus* were cultured in MRS broth (BD Difco). Other strains were cultivated in Chopped meat broth media supplemented with hemin, menadione, and vitamin K_1_ (MBcell).

### Quantification of SCFAs

The culture supernatant was obtained by centrifugation at 6,000 rpm and 4°C and was then concentrated twice using a vacuum concentrator (Eppendorf Concentrator plus, Germany). The sample was filtered through a 0.45 μm syringe filter and subsequently analyzed using high-performance liquid chromatography (HPLC; Agilent 1260, Agilent Technologies, USA). For the separation and quantification of organic acids and SCFA, including lactic acid, acetic acid, propionic acid, and butyric acid, an Aminex HPX-87H column (300 mm × 7.8 mm, 9 μm particle size; Bio-Rad Laboratories, USA) was used. The mobile phase consisted of 0.005 N sulfuric acid with a flow rate of 0.6 ml/min. The column temperature was maintained at 60°C, and a 10 μl sample was injected. Peaks were detected using both a UV detector and a refractive index detector (RID) set at 210 nm. The amount of SCFAs produced by each strain was calculated by subtracting the concentration in the culture supernatant from that of the GMM blank. For quantification of SCFAs, the external calibration curve was calculated by the analysis of standards at following concentration levels: 0.3125, 0.625, 1.25, 2.5, 5, and 10 mM, and the limit of detection (LOD) was in the range of 0.09 to 0.23 mM.

### Quantification of B Vitamins

The culture supernatant was concentrated using a vacuum concentrator (Vision VS-802, VISIONBIONEX, Republic of Korea) for 24 h at room temperature. The concentrated samples were re-dissolved in mobile phase A, concentrated ten-fold, and then filtered through a 0.2 μm syringe filter prior to HPLC analysis (Shiseido Nanospace Sl-2, Shiseido, Japan). For the analysis and quantification of vitamins, including B_1_, B_2_, B_3_, B_6_, B_9_ and B_12_, a CapCellPAK 120UG C18 column (Osaka Soda, Japan) was used. Mobile phase A was prepared by adding 10 ml of PIC (paired-ion chromatography) reagent to 500 ml of ultrapure water, and then filtering it through a 0.2 μm syringe filter after degassing. Mobile phase B was prepared by adding 10 ml of PIC reagent to 500 ml of 60%methanol and filtering through a 0.2 μm syringe filter after degassing. The PIC reagent was formulated by dissolving 1 g of 1-heptanesulfonic acid (Sigma) in a solution of 10 ml of distilled water and 10 ml of acetic acid (Sigma). The flow rate was set at 0.5 ml/min with the following gradient elution: 0-3 min, 5% B; 3–4min, 13% B; 4-12min, 20%B; 12–15min, 25% B; 15-17min, 30% B; and 17–20min, 33% B; 20-24 min, 40% B; 24-30 min, 45% B; 30-32 min, 45% B; 32-35 min, 40% B; 35-37 min, 40% B; 37-45 min, 60% B; 45-50 min, 100% B; 50-55 min, 5% B. The column temperature was consistently held at 40°C. For each analysis, 20 μl of the sample was injected, and peak detection occurred at 270 nm using a UV detector. The amount of B vitamins produced by each strain was calculated by subtracting the concentration in the culture supernatant from that of the GMM blank. For quantification of B vitamins, the external calibration curve was calculated by the analysis of standards at following concentration levels: 31.25, 62.5, 125, 250, and 500 μg/l, and the LOD was in the range of 17 to 35 μg/l.

### Metabolome Analysis

Probiotic culture supernatant (750 μl) was diluted in 2.25 ml of ice-cold methanol and vortexed for 1 min, followed by centrifugation at 13,000 g for 10 min at 4°C. One hundred microliters of supernatant was collected, concentrated to dryness in a vacuum concentrator, and stored at -80°C until required. Samples were derivatized by adding 30 μl of a solution of 20 mg/ml methoxyamine hydrochloride in pyridine (Sigma, USA) for 90 min at 30°C, and then adding 50 μl of N, O-bis(trimethylsilyl)trifluoroacetamide (BSTFA; Sigma) and heating for 30 min at 60°C. A mixture of alkane standards and fluoranthene was used as retention indices and an internal standard, respectively. GC-MS analysis was conducted using a Thermo Trace 1310 GC (Thermo, USA) coupled to a Thermo ISQ LT single quadrupole mass spectrometer (Thermo). GC was performed using a DB-5MS column (60-m length, 0.25 mm i.d., and 0.25-μm film thickness) (Agilent). Derivatized samples were injected at 300°C using a split ratio of 1:5, and metabolites were separated using a helium flow of 1.5 ml using the following oven program; 2 min at 50°C, 50°C to 180°C at 5°C/min, 8 min at 180°C, 180°C to 210°C at 2.5°C/min, 210°C to 325°C at 5°C/min, and 10 min at 325°C. Mass spectra were acquired in the scan range 35-650 m/z at 5 spectra per sec in electron impact ionization mode and an ion source temperature of 275°C. Spectra were processed using Thermo Xcalibur and AMDIS software with automated peak detection, and metabolites were identified by matching mass spectra and retention indices using the NIST Mass spectral search program (version 2.0, USA) and MS-DIAL (http://prime.psc.riken.jp/compms/msdial/main.html). Relative metabolite intensities were normalized by the sum of identified peaks.

### Co-Culture Experiments

A single colony of each strain was grown in its strain-specific medium for 72 h and was then washed three times with 0.85% (w/v) NaCl to remove impurities. The cells were diluted to an OD_600_ of 0.1 in fresh modified SHIME (mSHIME) medium, which mimics the physiological condition of the human gut environment. Pairwise co-cultures were established by pooling the diluted cultures in a 1:1 ratio. For monocultures, only a single bacterial culture was used. Folate-deficient mSHIME comprised 1.2 g/l arabinogalactan, 2 g/l pectin, 0.5 g/l xylan, 0.4 g/l glucose, 3 g/l yeast extract, 1 g/l peptone, 2 g/l mucin, 0.5 g/l cysteine-HCl, 4 g/l starch, 1 ml/l Tween 80, 1 ml/l Microelement solution, and 1 ml/l Vitamin solution. The Microelement solution contained 500 mg/l MnSO_4_, 100 mg/l FeSO_4_, 100 mg/l CoSO_4_, 100 mg/l ZnSO_4_, 10 mg/l CuSO_4_, 10 mg/l Alk(SO_4_), 10 mg/l H_3_BO_3_, 100 mg/l Na_2_MoO_4_, 100 mg/l NiCl_2_, and 10 mg/l Na_2_SeO_3_. Vitamin solution included 1 mg/l menadione, 2 mg/l biotin, 10 mg/l pantothenate, 5 mg/l nicotinic acid, 0.5 mg/l vitamin B_12_, and 4 mg/l thiamine. The cells were grown in an anaerobic chamber with 10% CO_2_, 5% H_2_, and 85% N_2_. Growth was monitored by measuring OD_600_ and CFU counting.

### Statistical Analysis

Experimental data from all studies were evaluated by Student's *t*-test and One-way ANOVA using GraphPad Prism 10 (USA). Differences were considered significant when *p* or P values were below 0.05. Multivariate analysis was performed using MetaboAnalyst 5.0 (https://www.metaboanalyst.ca/) [21].

## Results

### Characterization of SCFAs Produced by Selected Probiotic Strains

**Fig. 1:**
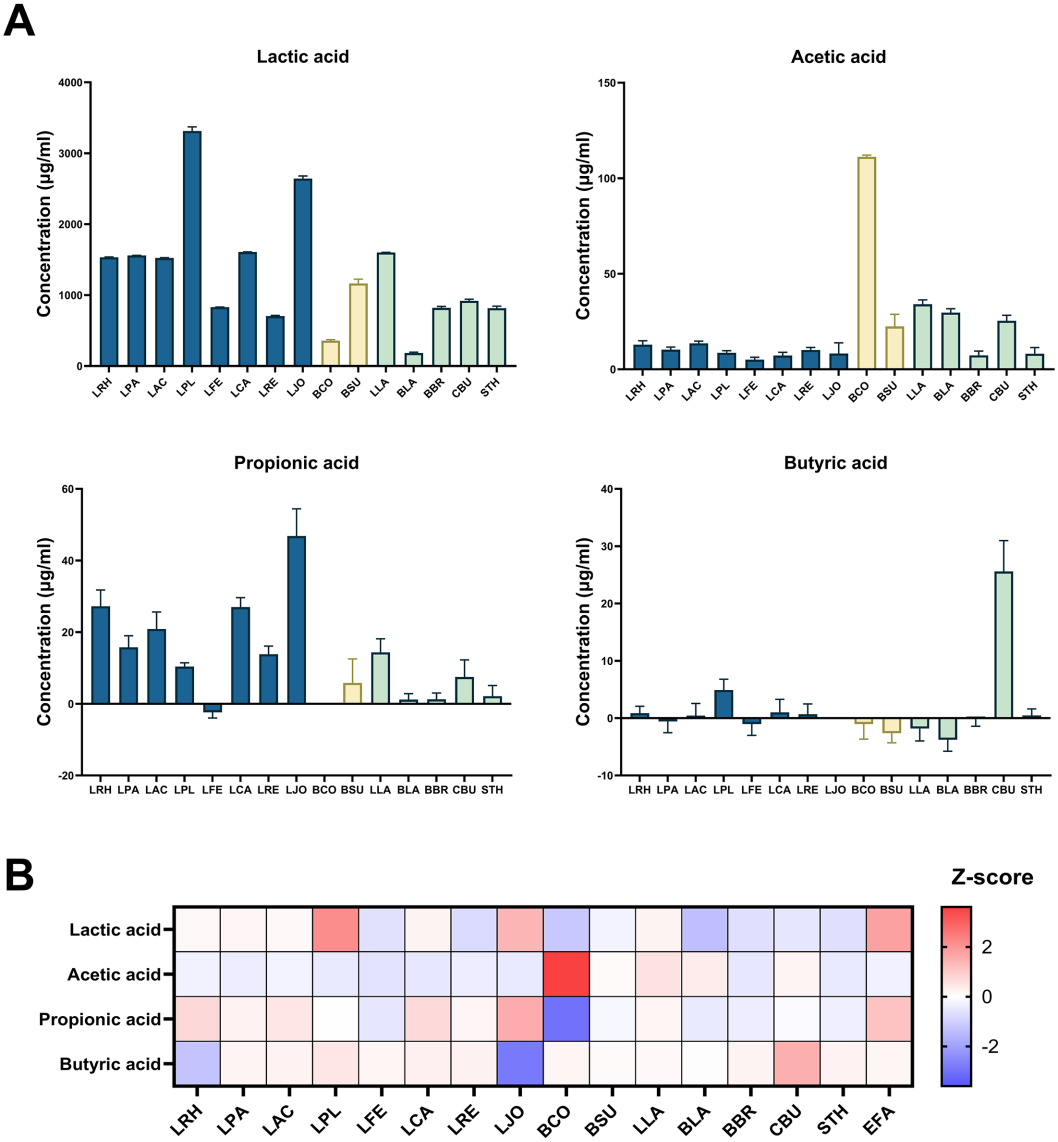
Analysis of SCFA production by probiotics. (**A**) Production of SCFAs by individual probiotic strains. Bacterial strains belonging to the families of *Lactobacillaceae*, *Bacillaceae*, and others are indicated in different colors. The amount of SCFAs produced by each strain was calculated by subtracting the concentration in the culture supernatant from that of the GMM blank. Data are expressed as mean ± standard deviation. (**B**) Heatmap representation of SCFAs production across 16 probiotic strains. Metabolite concentration was normalized using the Z-score, which is the raw score minus the population mean, divided by the population standard deviation. Abbreviations of each strain are indicated in Table 1.

SCFAs are one of the microbial fermentation products involved in central carbon metabolism, which have various impacts on host physiology [16]. These molecules are known to serve as energy substrates for intestinal cells and as signaling molecules associated with the regulation of inflammation and tumorigenesis. To evaluate the SCFAs production profiles of 16 probiotic species, we measured lactic, acetic, propionic, and butyric acid concentrations in their culture supernatants grown in GMM medium. As expected, a number of strains producing lactic acid were involved in the family of *Lactobacillaceae*, especially high in *Lactiplantibacillus plantarum* and *Lactobacillus johnsonii* (Fig. 1). Production of acetic, propionic and butyric acids were highest in *Bacillus coagulans*, *L. johnsonii* and *Clostridium butyricum*, respectively, and *Lactobacillaceae* showed greater yield in propionic acid compared to other strains. Butyric acid production was found solely in *C. butyricum* and *L. plantarum* with a lesser amount. In summary, SCFAs production depends on the type of probiotic species. While *Lactobacillaceae* produced higher amount of lactic acid and propionic acid among the probiotic strains, non-lactic acid producing bacteria such as *Bacillus* and *Clostridium* showed remarkable acetate and butyrate production compared with other genera.

### Characterization of B Vitamins Produced by Selected Probiotic Strains

**Fig. 2:**
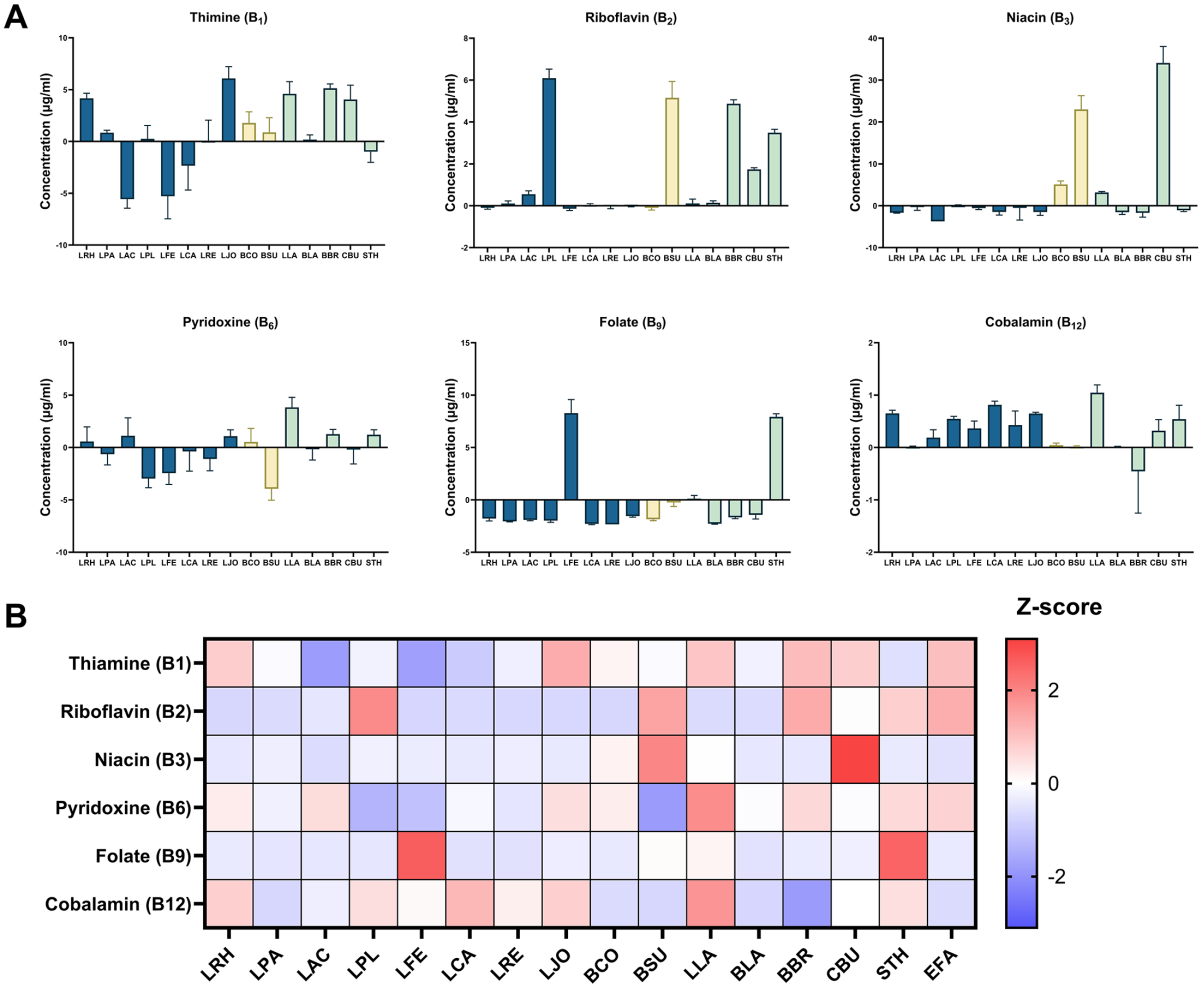
Analysis of B vitamin production by probiotics. (**A**) Production of B vitamins by individual probiotic strains. Bacterial strains belonging to the families of *Lactobacillaceae*, *Bacillaceae*, and others are indicated in different colors. The amount of B vitamins produced by each strain was calculated by subtracting the concentration in the culture supernatant from that of the GMM blank. Data are expressed as mean ± standard deviation. (**B**) Heatmap representation of B vitamins production across 16 probiotic strains. Metabolite concentration was normalized using the Z-score, which is the raw score minus the population mean, divided by the population standard deviation. Abbreviations of each strain are indicated in Table 1.

**Fig. 3:**
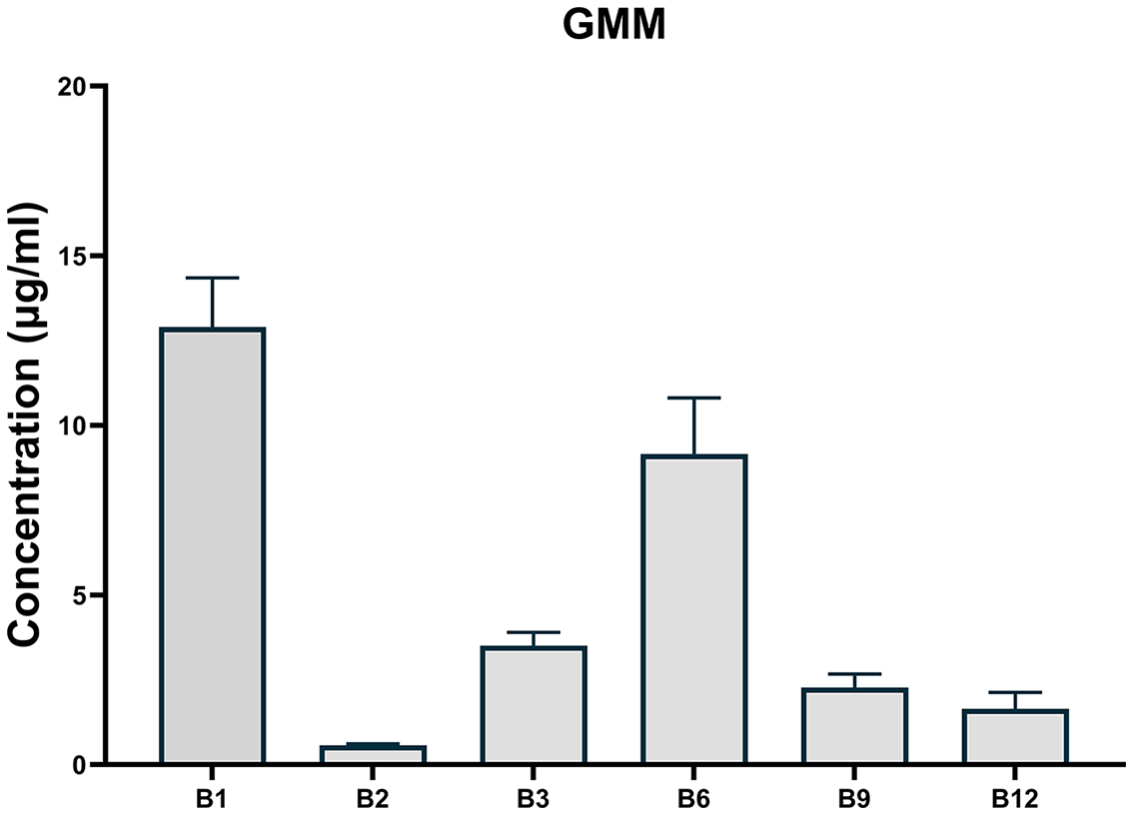
Concentration of B-vitamins present in the control GMM medium.

B vitamins are crucial cofactors for human body function, including fat, protein, and carbohydrate metabolism as well as DNA synthesis [22]. Human cells obtain these nutrients either from diets or the gut microbiota. Probiotics are generally known to provide B vitamins promoting human health; however, integrative information on B vitamins production profiles of all probiotic species is limited. We measured concentrations of six B vitamins, B_1_ (thiamine), B_2_ (riboflavin), B_3_ (niacin), B_6_ (pyridoxine), B_9_ (folate) and B_12_ (cobalamin), in culture supernatants and compared the production profiles among the probiotic species. As shown in Fig. 2, biosynthesis of B vitamins was distinctive for specific species. For example, biosynthesis of both B_2_ and B_3_ was high in *Bacillus subtilis* and *C. butyricum*, and these vitamins were also produced by *L. plantarum*, *B. breve*, and *Streptococcus thermophilus* for B_2_ and *B. coagulans* for B_3_, respectively. Vitamin B_9_ was only produced by *Limosilactobacillus fermentum* and *S. thermophilus*. It is noted that the control GMM medium already contained some vitamins including B_1_, B_3_, B_6_ and B_9_, and the concentration of these vitamins was negative in specific probiotic species, depending on their requirements for growth (Fig. 3). In summary, B vitamin production depends on the type of probiotic species and possibly on the strain level, suggesting that probiotic supplementation can be precisely personalized according to an individual’s nutritional status.

### Characterization of Global Metabolites Produced by Selected Probiotic Strains

**Fig. 4:**
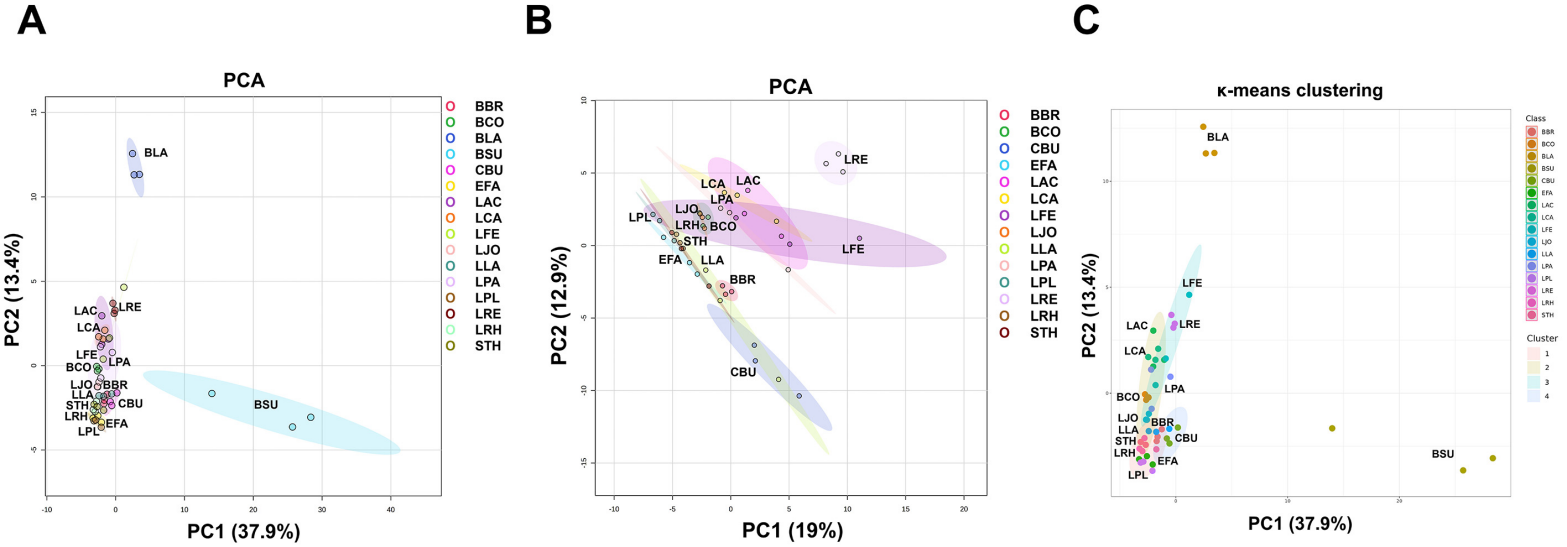
Analysis of global metabolites produced by probiotics. Metabolic profiles of culture supernatants of probiotics grown for 24 h were analyzed using principal component analysis (PCA, **A and B**) and *k*-means clustering (**C**) with auto-scaling. (**A**) A score plot of PCA analysis of 16 probiotic strains. (**B**) A score plot of PCA analysis of 14 probiotic strains, excluding BLA and BSU, which exhibited distinct metabolic profiles compared to the others. (**C**) Clustered groups generated by *k*-means clustering algorithm. Abbreviations of each strain are indicated in Table 1.

Each probiotic strain has been distinguished with precisely defined effects on human health. Microbe-derived metabolites are one of the main effectors, and recent studies have focused on the discovery of these functional metabolites [6, 7, 23]. To identify the metabolic features of widely used commercial probiotics, we analyzed their global secreted metabolite profiles of 16 probiotic strains (Fig. 4). A total of 105 metabolites were identified, covering sugars, amino acids, polyamines, organic acids, and fatty acids across all the probiotics. Unsupervised principal component analysis (PCA) and *k*-means clustering showed clustered probiotics into 4 groups with exceptions for *B. lactis* (BLA) and *B. subtilis* (BSU), which had distinct metabolic profiles compared to other strains (Fig. 4A).

**Fig. 5:**
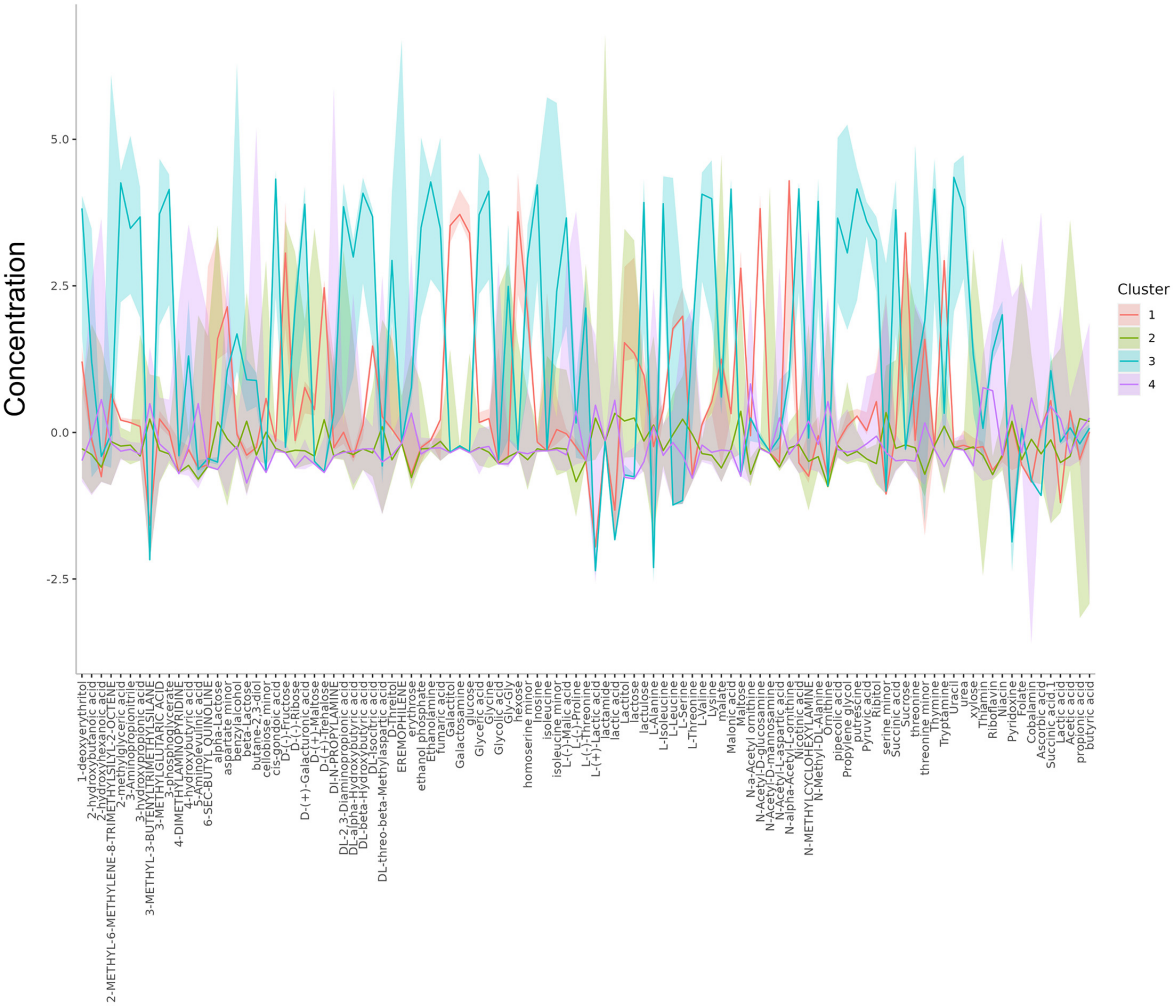
List of metabolites unique to the cluster 1 to 4. Cluster 1, red; Cluster 2, green, Cluster 3, blue; and Cluster 4, purple, respectively.

**Fig. 6:**
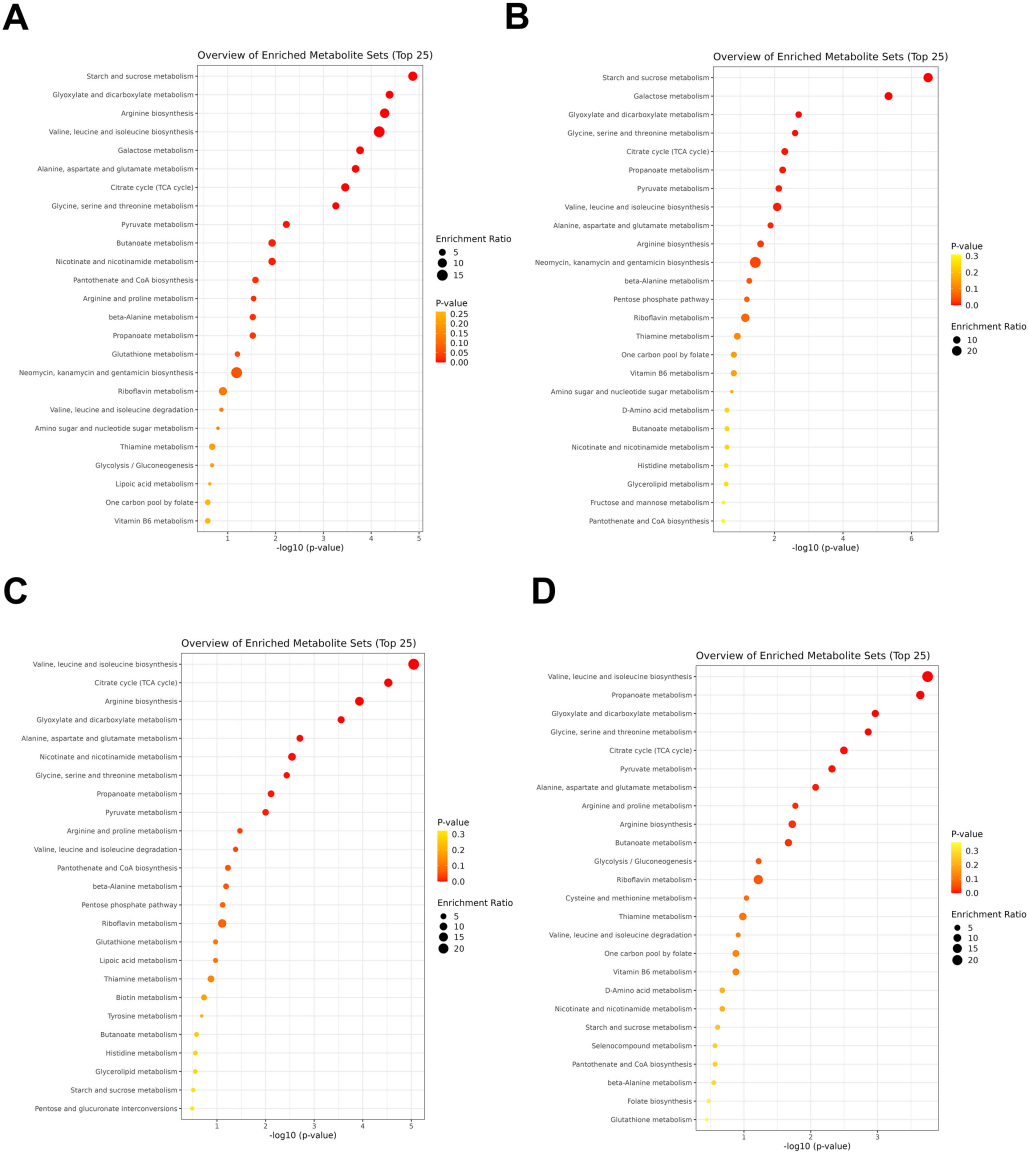
Pathway analysis based on the enriched metabolites in each cluster, identifying the most relevant metabolic pathways via pathway impact and adjusted *p*-value. The figures were generated using MetaboAnalyst (www.metaboanalyst.ca). The circle size indicated the enrichment ratio of the pathway, while the color represents the significance in red.

The probiotic strains were grouped similarly by both algorithms of PCA and *k*-means clustering, primarily containing clusters 1 and 2 with *S. thermophilus*, *E. faecium*, *L. plantarum*, *L. rhamnosus*, *L. acidophilus*, *L. casei*, and *B. coagulans*, and cluster 3 with *L. reuteri* and *L. fermentum*, while cluster 4 consisted of *C. butyricum* (Fig. 4 and Table 2). Specific metabolites contributing to each clustered group are indicated in Table 2 and Fig. 5. Additionally, important metabolic pathways associated with the metabolites demonstrated the unique metabolic properties of each cluster (Fig. 6). Clusters 1 and 2, comprising various genera from *Streptococcus* to *Bacillus*, tended to produce more sugars and amino sugars compared to other clusters, thereby impacting starch and sugar metabolism. The probiotic strains in cluster 3 were specialized as *Lactobacillaceae* and uniquely produced various organic acids, amino acids and polyamines. In particular, metabolite production involved in nicotinate and nicotinamide metabolism, and pantothenate and CoA biosynthesis were distinct in this group, supporting the importance of this family group as providers of physiologically bioactive compounds. Cluster 4 reflected mainly the metabolic profile of *C. butyricum*, characterized by the production of cobalamin, ornithine, and 2-hydroxyhexanoic acid. Our results suggest that each probiotic strain has a unique metabolic profile, and the selection of specific probiotic panels based on their secreted metabolite features would provide more precise health benefits.

### Folate Production by *S. thermophilus*

**Fig. 7:**
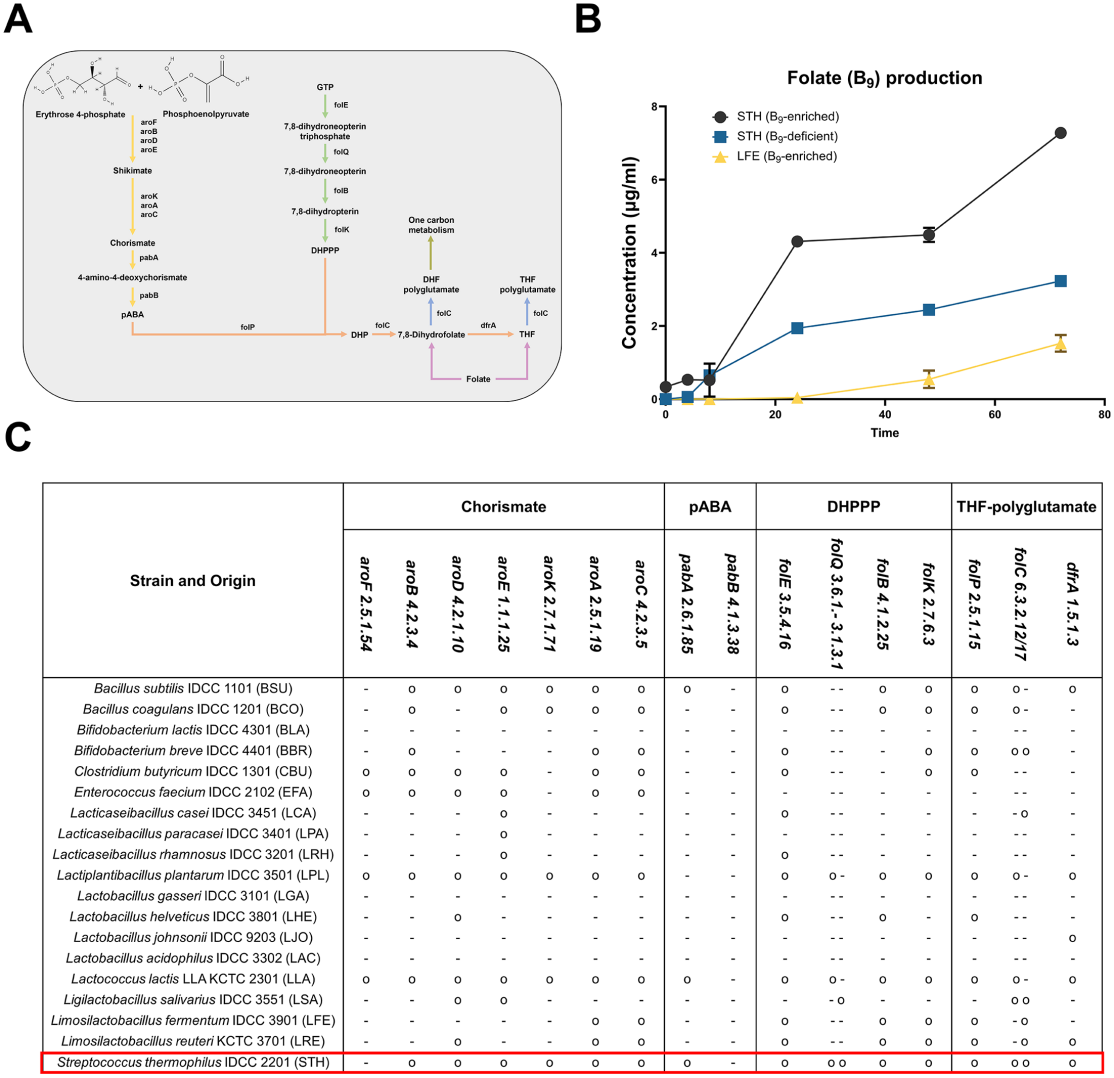
Folate production by *S. thermophilus*. (**A**) Representative folate biosynthetic pathway. (**B**) Production of folate (vitamin B_9_) of *S. thermophilus* and *L. fermentum* in a conditional folate environment. STH, *S. thermophilus*; and LFE, *L. fermentum*. (**C**) Genes and enzymes for the folate biosynthesis predicted from the sequenced genomes of probiotic strains. pABA, para-aminobenzoic acid; DHPPP, 6-hydroxymethyl-7,8-dihydropterin pyrophosphate; and THF, tetrahydrofolate.

Folate, vitamin B_9_, is involved in several cellular functions such as de novo nucleotide synthesis, global methylation, and amino acid synthesis [24, 25]. Among the probiotic species, only *L. fermentum* and *S. thermophilus* showed significant production of folate compared with the GMM control (Fig. 2). To determine essential genes required for folate biosynthesis, we analyzed the presence of genes from each bacterial genome encoding specific enzymes in the defined folate biosynthesis pathway (Fig. 7). Compared with other species, *S. thermophilus* possesses entire gene sets required for biosynthesis of DHPPP ([7,8-dihydropterin-6-yl] methyl-diphosphate) and poly-glutamylation of tetrahydrofolate. In vitro growth experiment of *S. thermophilus* in the mSHIME mimicking human intestinal environment further verified production of folate secreted by *S. thermophilus*, while less amount was produced by *L. fermentum* (Fig. 7B). Overall, each probiotic strain exhibited a unique B vitamin biosynthesis profile, and especially, *S. thermophilus* was suggested as a potential folate provider to host as well as commensal gut microbiota for nutrient cross-feeding.

### Interaction of *S. thermophilus* with Gut Commensal Bacterial Species

**Fig. 8:**
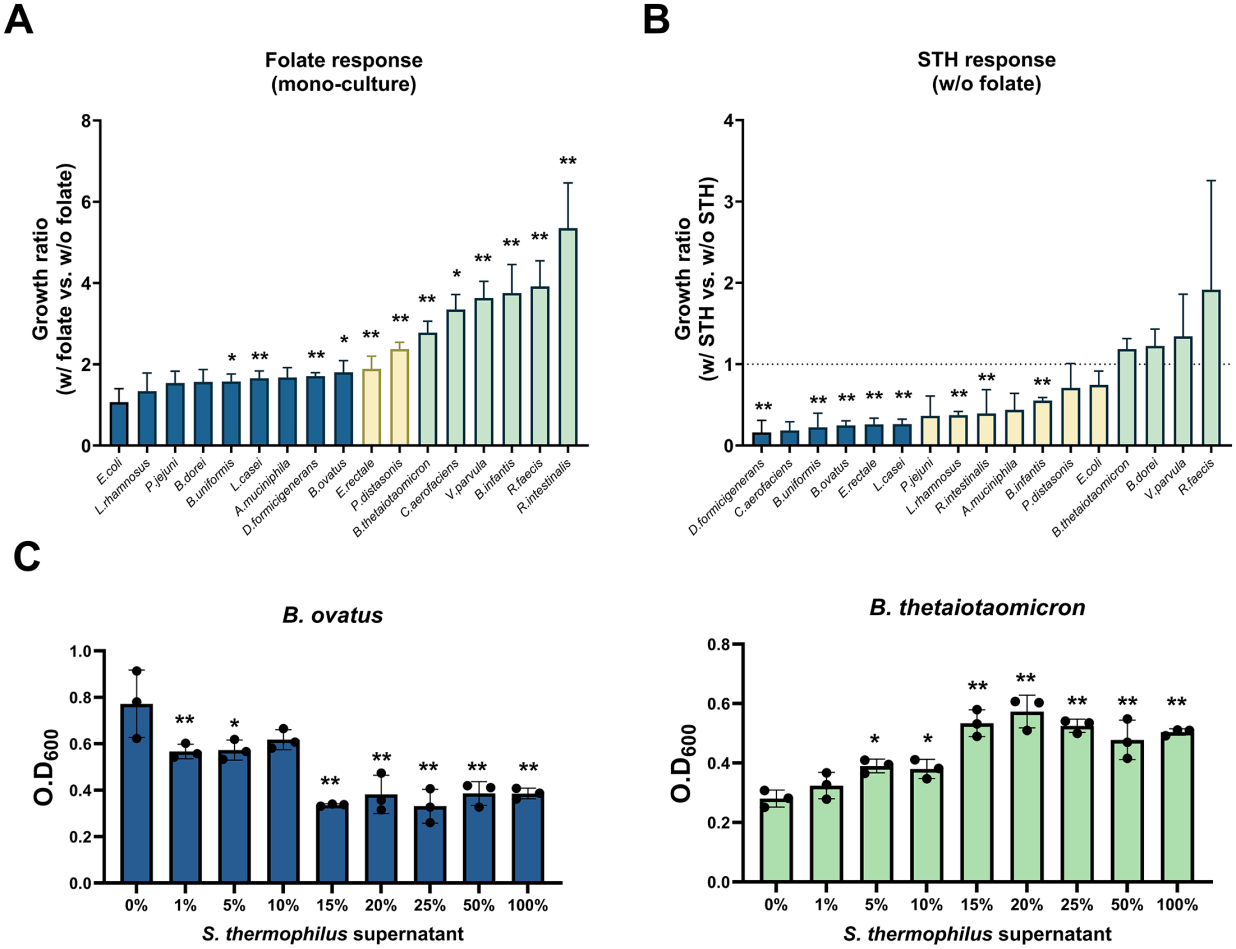
Bacterial growth dependent on folate and *S. thermophilus*. (**A**) Ratio of individual bacterial growth comprising the human gut microbial community in the folate-enriched medium versus in the folate-deficient medium. (**B**) Ratio of individual bacterial growth comprising the human gut microbial community when co-cultured with *S. thermophilus* versus when mono-cultured on the folate-deficient medium. Significant differences between the two conditions are indicated by asterisks at the 95% (*) and 99% (**) significance levels using Student’s *t*-test. (C) Growth of *B. ovatus* and *B. thetaiotaomicron* with supplementation of *S. thermophilus* supernatant. Significant differences among the conditions are indicated by asterisks at the 95% (*) and 99% (**) significance levels using one-way ANOVA with Dunnett’s post-hoc analysis.

More than 20% of human gut microbial communities are predicted to be auxotrophic species. Their viability is dependent on acquiring B vitamins from prototrophic microbes, such as *S. thermophilus*, as indicated in this study [26]. Folate deficiency is known not only to alter gut microbiota composition but also to disrupt intestinal integrity and cause persistent diarrhea [27, 28]. To investigate the effects of folate deficiency on the growth of commensal gut bacteria, we first selected 17 bacterial species that are largely abundant in human gut microbiota and evaluated their folate responses. As shown in Fig. 8A, each bacterial species exhibited different growth dependency on folate. When folate was present, *B. thetaiotaomicron*, *V. parvula*, *R. faecis*, and *R. intestinalis* grew better compared to the absence of folate. Conversely, *E. coli*, *L. rhamnosus*, *P. jejuni* and *B. dorei* were not responsive to folate.

Given that *S. thermophilus* has been suggested as a potential folate provider to the commensal gut microbiota, we performed a co-culture experiment with *S. thermophilus* in the absence of folate in relation to folate deficiency. Many strains demonstrated a negative interaction with *S. thermophilus* (Fig. 8B). No bacterial strain showed a significantly positive interaction with *S. thermophilus* in co-culture compared to its mono-culture in the folate-deficient media. However, compared to other species showing inhibited growth by co-culture with *S. thermophilus*, several bacteria, including *B. thetaiotaomicron*, *V. parvula*, and *R. faecis*, were not affected. Interestingly, these bacterial species also exhibited greater folate dependence than other bacteria, suggesting that they may obtain growth advantages in the interaction with *S. thermophilus* through folate produced by the bacterium.

To assess whether the observed growth promotion results from factors such as nutrient competition rather than direct folate dependence, we compared the growth of specific bacterial species, including *B. ovatus* and *B. thetaioaomicron*, which differ in their dependence on folate and *S. thermophilus*. As shown in Fig. 8C, *B. thetaiotaomicron* exhibited dose-dependent growth enhancement with the addition of *S. thermophilus* supernatant, while the growth of *B. ovatus* decreased upon treatment. This result suggests that nutrient competition is unlikely to be responsible for the observed growth change. However, *S. thermophilus* supernatant contains various bioactive molecules that may influence bacterial growth beyond folate, necessitating further sophisticated investigations. Although detailed analysis of the mechanism of interactions between *S. thermophilus* and gut bacteria is required, our results imply that specific bacteria are dependent on folate, and supplementation of *S. thermophilus* can affect their growth by providing folate under conditions of folate deficiency.

## Discussion

Precision probiotics refer to specific bacterial strains with precisely defined effects. One of the microbial factors that confer these probiotic effects is the metabolites derived from microbes, such as SCFAs and B vitamins. In this study, we evaluated the metabolic profiles of widely used probiotic strains and identified *S. thermophilus* as a key producer of folate. In vitro co-culture experiments with *S. thermophilus* and individual species comprising the human gut microbial community revealed that the growth of *B. thetaiotaomicron*, *V. parvula*, and *R. faecis* were dependent on both folate and *S. thermophilus*.

Folate plays a role in numerous metabolic functions, including nucleic acid biosynthesis, amino acid metabolism, and universal methylation [15]. Recent studies have reported on the roles of microbe-derived folate in host health [29, 30]. For instance, Qiao *et al*. discovered that *Bacteroides spp*. are major contributors to gut folate biosynthesis, and this contributes to the amelioration of hepatic steatosis [29]. Thomas *et al*. demonstrated that bacterial folate metabolism, mediated by FolC2, suppresses inflammation [30]. It is speculated that microbial folate can alter host folate and one-carbon metabolism, which is interconnected with other central metabolic processes, such as epigenetic modifications and DNA repair [31]. Conventionally, lactic acid bacteria such as *Bifidobacteria* and *Lactobacilli* are recognized as prominent folate producers. However, folate biosynthesis appears to be limited to specific species and strains [15]. Systemic approaches, including genome assessment, have been used to predict the distribution of folate synthetic pathways among human gut microbes. Nonetheless, there are limitations due to discrepancies between the predicted results and actual production [22].

In the current study, we identified *S. thermophilus* as the most effective folate provider among 16 popular probiotic strains. *S. thermophilus* has long been used as a starter culture in dairy products, making it the second most important species of industrial lactic acid bacteria after *L. lactis* [32]. It is also well-known for its production of folate, which is associated with anti-cancer and anti-oxidative activities [32, 33]. To synthesize folate in bacteria, two metabolic branches combine to generate dihydropteroate: pABA (p-aminobenzoic acid) and DHPPP (6-hydromethyl-7,8-dihydropterin) branches. It has been suggested that dihydroneopterin synthesis, encoded by folQ, may be the metabolic bottleneck in *S. thermophilus* [33]. Although the catalytic mechanism of folQ is yet clearly defined, it is believed to mediate the removal of pyrophosphate from DHNTP (dihydropterin triphosphate), a step that is often absent in many other bacterial species.

While there have been comprehensive studies on streptococcal functions and metabolic characteristics, only a few reports have addressed its interaction with the gut microbiota [34, 35]. In mice induced with colorectal cancer, oral administration of *S. thermophilus* increased the abundances of *Bifidobacterium* and *Lactobacillus* by secreting β-galactosidase, which has anti-cancer effects [35]. In septic mice, the administration of *S. thermophilus* alleviated inflammation caused by sepsis, leading to an increase in *Fusobacterium* and reduction of *Flavonifractor* [34]. However, these studies focused on specific disease states and lacked an in-depth exploration of individual bacterial interactions. In our study, we evaluated microbe-microbe interactions, consisting of human gut microbiota, and identified *V. parvula*, *B. thetaiotaomicron*, *R. faecis* as folate-responsive species that also associated with folate produced by *S. thermophilus*.

*V. parvula* is a Gram-negative coccus usually found in the oral flora. It is also present in the gastrointestinal tract as part of the normal flora in healthy individuals, yet is associated with several diseases including oral cavities, endocarditis, and ulcerative colitis [36-38]. As part of the oral flora, the interaction of *V. parvula* with *Streptococcus* spp. has been highlighted in several studies [39-41]. In particular, certain *Streptococcus* species can provide lactate to *V. parvula*, promoting the formation of multi-species biofilms, which lead to periodontitis and dental caries [41]. Besides lactate, the authors speculated that other factors could influence the interaction between these microbial genera. In our study, *V. parvula* showed better growth with *S. thermophilus*, compared to other bacteria under the condition of folate deficiency. The reasons behind the synergistic growth of both strains in the absence of folate remains speculative, but one theory posits that folate-responsive factors from *S. thermophilus* might stimulate *V. parvula*‘s growth.

*B. thetaiotaomicron* is one of the most common bacteria found in the human gut and has been extensively studied as a symbiont due to its polysaccharide-metabolizing abilities [42, 43]. This organism breaks down dietary fiber polysaccharides and host glycans while modulating the host immune system [44]. Fermentation of polysaccharides by *Bacteroides* also provides nutrients like volatile fatty acids for other intestinal bacteria [45]. We observed that *B. thetaiotaomicron* positively interacts with *S. thermophilus* in the absence of folate. Beyond its unique glycan degradation capabilities, *Bacteroides* is recognized as a significant folate producer in the gut, influencing liver metabolism by mitigating nonalcoholic hepatic steatosis [29]. Although the growth-promotion mechanism of *B. thetaiotaomicron* by *S. thermophilus* is yet to be fully understood, it is evident that their interaction can enhance gut microbial B-vitamin synthesis, beneficial to folate-deficient patients. Similar to *B. thetaiotaomicron*, *R. faecis* represented a positive interaction with *S. thermophilus* in the absence of folate. *R. faecis*, one of the 12 most dominant bacterial species in the large intestine, degrades complex polysaccharides in the colon, producing butyrate that influences various cellular functions crucial for host colonic health [46, 47]. Both *B. thetaiotaomicron* and *R. faecis*, as glycan degraders and butyrate producers, showed higher growth with *S. thermophilus* in the context of folate deficiency. Comprehensive transcriptomic and metabolic experiments regarding the effect of *S. thermophilus* on these strains are needed to elucidate the connections among polysaccharide fermentation and folate production.

The findings that *S. thermophilus* promotes the growth of specific bacterial families are supported by several *in vivo* studies. For example, supplementation with *S. thermophilus* strains has been shown to increase the relative abundance of *Ruminococcaceae* in C57BL/6J mice [48]. Additionally, microbiome analysis results indicate that when *S. thermophilus* was administered, *Bacteroidaceae* significantly increased in Sprague-Dawley rats [49]. Veillonella species have also been observed to commonly co-exist with *S. thermophilus* in the intestine, potentially due to their metabolic interactions [50]. These findings suggest that *S. thermophilus* can influence the growth of specific bacterial groups; however, more comprehensive studies investigating its *in vivo* effects are still needed.

While our study suggests a potential interaction between *S. thermophilus* and gut bacterial species via folate production, it has certain limitations. Pairwise co-culture experiments were employed to facilitate a clearer understanding of microbe-microbe interactions; however, this system may not fully capture the complexity of higher-order interactions involving multiple species. In addition, although folate biosynthesis by gut microbiota is generally considered beneficial to the host, excessive folate production may have potential drawbacks. These include disruptions in folate homeostasis, imbalances in gut microbiota, promotion of cancerous cell growth, and exacerbation of inflammatory conditions [51-53]. A continuous supply of bacterial folate to the colon may elevate host folate levels, potentially altering folate homeostasis and associated metabolic pathways [53]. Folate plays a dual role in cancer development; when present in excess, it can promote rapid cell growth and disrupt epigenetic modulation. Several studies have linked increased bacterial folate synthesis to diseases such as inflammatory bowel disease, small intestinal bacterial overgrowth, and aging [54-55]. Future studies are needed to further clarify bacterial folate synthesis and its effects on host metabolism.

## Conclusion

Our findings confirm that probiotics possess distinct metabolic profiles, including SCFAs and B vitamins. Moreover, the pairwise co-culture of probiotics with human gut microbiota reveals specific microbe-microbe interactions driven by nutrient cross-feeding, with a notable emphasis on folate. These results suggest that each nutrient cross-feeding interaction could represent a microbial survival strategy in response to environmental limitations. This underscores the importance of understanding interactions between human gut microbes and their metabolic profiles.

## Figures and Tables

**Table 1:** Bacterial strains used in this study.

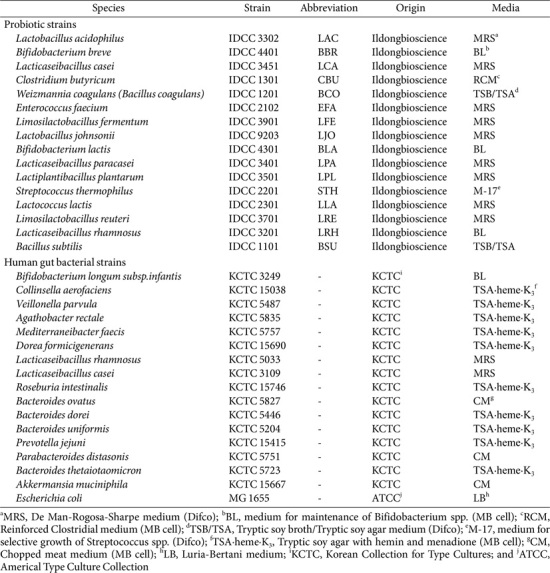

**Table 2:** Specific metabolites associated with each cluster of 16 probiotic strains.